# Anthropogenic pollutants – an insidious threat to animal health and productivity?

**DOI:** 10.1186/1751-0147-54-S1-S2

**Published:** 2012-02-24

**Authors:** Stewart M Rhind

**Affiliations:** 1 The James Hutton Institute, Craigiebuckler, Aberdeen, AB15 8QH, UK

## Abstract

**Summary:**

Humans have always polluted their environment and, to an extent, the associated adverse consequences have increased in parallel with the global population. However, in recent decades, entirely novel compounds have been created, for multiple purposes, and some of these have become ubiquitous, damaging pollutants, which interfere with fundamental physiological processes in all animal species, disrupting reproductive and other functions. Understanding of the actions of these chemicals is poor but it is recognised that they can act additively, at low concentrations, and that animals at early stages of development are particularly sensitive to their effects. All species, including domestic and wild animals and humans, can be affected. Thus, there are potential adverse implications of exposure for farm and companion animal productivity and health, and associated economic implications. While anthropogenic pollutants exert subtle, but important, adverse effects on animal health and productivity, these should be weighed against the benefits associated with the use of these compounds, particularly in relation to food production and short-term determinants of animal health. However, it is suggested that it may be necessary to regulate future production and use of some of these compounds in order to ensure long term sustainability of production systems.

## Introduction

Pollution is a natural phenomenon. For example, crude oil constantly leaks from below the Earth’s crust in many areas, volcanic outputs include multiple toxic gases and forest fires result in the production of toxic hydrocarbons. However, both the amounts and range of types of pollutants released into the environment have been greatly increased by human action since metals were first smelted, thousands of years ago, increasing environmental levels of heavy metals [1]. The increases have been particularly marked during the last 60 years, as many new organic chemicals have been synthesised and used in thousands of products. The recently-synthesised compounds are chemically-diverse in nature, each having been created to have certain properties e.g. organochlorine compounds such as DDT are highly effective insecticides, phthalates are used as softening agents in plastics, polybrominated diphenyl ethers (PBDEs) are effective fire retardants used in electrical equipment and furnishings, and polychlorinated biphenyls (PCBs) were used, formerly, as coolants in electrical equipment [2]. Some others are by-products of combustion of the fossil fuels essential for modern lifestyles (polycyclic aromatic hydrocarbons; PAHs) [3,4]. All are found in the environment, although generally at very low concentrations.

The significance of this change in the nature and amount of pollutants lies in the fact that many of these chemicals have been found to perturb animal physiology, even although in most cases they are entirely synthetic and there is no naturally-occurring equivalent. Many of these compounds can bind to cellular receptors or otherwise interfere with hormonal signalling and enzyme systems in species as diverse as bacteria [5] and mammals [6]. Consequently, they have the capacity to disrupt normal endocrine function and are collectively described as endocrine disrupting compounds (EDCs). In addition to these organic pollutants, elemental pollutants, such as lead and mercury [7] also contribute to the pollutant burden and associated adverse physiological effects, sometimes acting in concert with organic pollutants [8].

Additional classes of anthropogenic compounds implicated in disruptive effects include some used to directly enhance animal and human health (analgesic pharmaceuticals; [9]) and crop production (nitrate fertilisers; [10]). All of these pollutants have become ubiquitous in the environment.

## Laboratory investigation of mechanisms and effects

Arguably, issues of environmental pollution and animal and human health first achieved prominence with the publication of Silent Spring [11]. This highlighted the effects of some of the new chemicals on wildlife and probably stimulated some of the many detailed studies of effects and mechanisms of action.

Many of the studies designed to elucidate mechanisms of action have involved laboratory rodents subjected to *high level*, *short-term*, *exposures* to *single chemicals*. Such studies have clearly shown that such exposure could induce structural and physiological changes in the reproductive system [12,13] and could compromise immune function [14,15]. Such studies also contribute to understanding of the underlying mechanisms of action of EDCs. In parallel with the laboratory studies, field observations of a wide range of species showed further evidence of adverse physiological effects, particularly in relation to reproduction [16,17] with associated effects on populations [18]. However, many of these observations concerned abnormally high levels of exposure associated with accidental releases of pollutants or with other unusual patterns of exposure (e.g. employment in factories where EDCs were used in manufacturing processes). Perhaps unsurprisingly, concerns about anthropogenic environmental pollutants remained relatively muted for some time because both field observations and laboratory studies involved high levels of single chemicals, making, it is easy to dismiss the observed effects as irrelevant in most circumstances. Environmental (low) concentrations of these compounds were seldom associated with *apparent* adverse effects on either animals or humans. Furthermore, many of the compounds concerned were of great value to crop production and disease control (herbicides and pesticides) or contributed to more comfortable (electrical equipment) or safer lifestyles (fire retardants) and the will to investigate possible adverse effects may have been limited.

## “Real world” exposure patterns are different

It is often suggested that low concentrations of environmental pollutants are of no concern because, in most circumstances, concentrations are below the No Observed Effect Levels (NOEL); i.e. environmental levels of each individual chemical have been shown to be below the minimum concentrations known to induce a physiological response. However, this argument fails to take account of several factors:

### a) Mixtures

It is now well recognised that EDCs act additively [19,20] and, possibly, synergistically [21] on physiological systems i.e. the combined effects of multiple EDCs, each of which is present at concentrations too low to induce a response, can induce adverse effects.

### b) Exposure differs with age and stage

It is often assumed that exposure to EDCs is similar in adult and fetal / juvenile stages of development and that they are subject to equivalent pollutant insults. In fact, studies of sheep have shown that fetal tissue concentrations of most EDC classes are lower than those of their dams, although some can be preferentially accumulated in fetal tissues [22,23]. This is presumably as a result of a shorter period of exposure in the fetus and differences between the fetus and dam in uptake, metabolism and excretion. In some animal groups, the insult may differ with stage of development because food sources, and therefore pollutant exposure patterns, differ e.g. immature ruminants feed on milk while mature animals are herbivorous; similarly, developing poultry derive nutrient from egg yolk while adults have very different food sources. These differences make it difficult to extrapolate tissue burden data across animals at different stages of development or to determine a critical level of exposure.

### c) Sensitivity differs with age and stage

The occurrence of lesser tissue EDC burdens in younger animals might be assumed to be associated with a lesser risk of disruption. However, like exposure rate, susceptibility to disruption by environmental pollutants differs with age being greater during early developmental stages [24]. Studies of sheep have shown that exposed fetuses exhibit disrupted development even although some equivalent effects are absent in their dams [25,26]. Thus, the NOEL is different for animals at different stages of development.

### d) Individuals and species differ

Individual animals exhibit a very high degree of variation in the rate of tissue accumulation of EDCs even when apparently exposed in an identical way; this presumably reflects differences between individuals in uptake, metabolism and excretion [2]. However, it should be noted that the capacity to metabolise pollutants is highly dependent, also, on species [27] and so extrapolation of the NOEL between species requires caution.

## Farmed animals

Initial concerns about EDCs were centred, primarily, on terrestrial, vertebrate wildlife, because it was in wild animals that adverse effects were first noted, but subsequent research has implicated EDCs in effects on farm animals [2], marine, freshwater and terrestrial invertebrates [28-30] and humans [31,32].

Modern husbandry practices and modern household environments are both associated with increased exposure to the ubiquitous EDCs through food, water, inhaled air and administered pharmaceuticals [33]. Interest in environmental pollutants with respect to animals reared for food or as pets lies in many different areas. Consumers of farm animal products are often concerned about possible health risks associated with contaminated meat and dairy products while producers are concerned with both the image associated with their products and its monetary value. Pet owners have concerns about their animal’s health and welfare. Consequently, veterinarians have an interest in all of these issues and species.

One aspect of exposure of farm animals that may change in the future is related to the increasing scarcity and cost of oil and phosphate, both of which are essential for the production of inorganic fertilisers; the increasing cost of such fertiliser, together with concerns about pollution, is increasing the pressure to recycle food waste, green waste and sewage sludge to land. All of these recycled products provide valuable plant nutrient but some also contain anthropogenic pollutants. There are divergent views with regard to the importance, in relation to animal productivity and health, of prolonged, low level, exposure to these pollutants following application of waste to land. In an extensive review, Smith [34] suggested that the application of sewage sludge to land was of little concern with respect to soil, animal or human health because rates of transfer into plants and animals were likely to be insignificant. Indeed, studies of tissue levels of several classes of EDCs (phthalate, PCBs, PBDEs and PAHs) suggest that exposure of sheep to pastures fertilised with sewage sludge resulted in minimal increases in milk or tissue concentrations of these compounds, relative to control animals exposed to inorganic fertilisers [35-38]. However, numerous, subtle, adverse effects have been observed in the same sheep [39]. While exposed animals appeared entirely normal, superficially, they exhibited many adverse changes in underlying physiology which had the potential to compromise reproductive performance. Specifically, exposure to sludge-treated pastures and associated EDCs has been shown to be associated with i) perturbed activity of several fetal hypothalamic neurotransmitter systems [25,26], ii) reduced fetal testis Leydig and Sertoli cell numbers and testosterone production [40] and associated germ cell numbers in the adult [41] and iii) increased fetal ovarian oocyte expression of the pro-apoptotic protein BAX and altered expression of many other fetal ovarian proteins [42]. In addition, preliminary data indicate that there may be disruption of maternal mammary structure [43] and altered protein expression in the fetal uterus [44].

Perturbation of non-reproductive systems has also been reported with sludge exposure being associated with reduced numbers of fetal thyroid follicles and reduced maternal T3 and T4 concentrations [45] and changes in offspring behaviour [46] and adult bone structure [47,48]. While not measured in the sheep studies, in other species effects of EDC exposure on obesogenic systems [49,50] and cardiovascular function have been recorded [51,52] and it is possible that similar disruption could occur in sludge-exposed ruminants or other animals exposed to an enhanced EDC insult.

In view of these observations, it might be expected that there would be evidence of reduced reproductive success in farmed animals as a result of increased environmental exposure to EDCs. At this time, evidence is scarce. Meijer et al. [53] reported a small reduction in fertility and milk production in dairy cows exposed to sewage-contaminated water and attributed the effect to the pollutants present. The high-yielding dairy cow has been the subject of much research because she exhibits a long-term decline in fertility but the underlying causes of this decline are not well understood. Nutritional and genetic factors have been implicated [54] but while, undoubtedly, they are involved, environmental pollutants may be acting in conjunction with them to exacerbate the decline in fertility through additive, subtle effects on gene expression and/or disruption of endocrine signals. The observations of Meijer et al. [53] may simply be indicative of a more general, largely invisible, effect of chronic, low level, environmental exposure to EDCs.

With regard to male animals, a decline in semen quality might be expected in the light of increased environmental exposure to EDCs and the effects on testis structure and function described above. One study of several farm species showed no reduction in sperm counts over a period of six decades [55]. However, it should be noted that the animals studied were selected for high fertility and may not be representative of the normal population. Furthermore, domestic ruminants store sperm and so may appear to have a high sperm count even when sperm production is reduced. Another study of bull semen [56] appeared to indicate a temporal decline in semen quality during the 1970s with an associated, anomalous improvement in sperm morphology and motility. It was concluded that there were some methodological inconsistencies which partially compromised the interpretation of the results but it was also concluded that the decline could not be readily linked to EDC exposure since semen quality subsequently improved. It seems likely that the issue is complex; it may involve environment/genotype interactions and a comprehensive understanding of the effects of environmental levels of EDCs on male fertility, or lack of them, may be some time off.

To date, little work has been done concerning effects of EDCs on domestic poultry but there is little reason to doubt that their physiology may be affected, also, since studies of wild birds have shown adverse effects of various EDCs on aspecets as diverse as egg shell formation and embryo survival [57,58] and brain function, as indicated by altered song patterns [15].

In holarctic regions, farming of animals for fur is common; these species (e.g. mink, arctic fox, etc) are carnivores, near to the top of the food chain, and accumulate relatively high concentrations of pollutants in their tissues; they too exhibit adverse effects on embryo and offspring survival when exposed to specific dietary EDCs [59].

Finally, a range of fish species are farmed throughout the world; some of them are exposed to higher rates of pollutant exposure than wild fish [60] because they are fed on products containing other fish and associated accumulated pollutants. While most concern is focussed on potential effects on human health, such elevated tissue burdens may have physiological / health consequences for the fish themselves. Consumers are concerned about tissue EDC accumulation but veterinarians may have concerns about potential reproductive or immuno-suppressive effects of EDC burdens. While there do not appear to be significant concerns about such effects, to date, it should be noted that effects of environmental pollutants on reproductive physiology [61] of wild fish species have been reported indicating potential susceptibility.

## Domestic pets

While no owner would wish to expose their much-loved companion animals to EDCs, as carnivores, dogs and cats are exposed to EDCs in their food [62]. Also, frequently they live indoors where EDC concentrations tend to be elevated relative to outdoor air [63] and so, like humans, they are exposed to a wide range of household EDCs, in part, owing to their close proximity to the ground where they are exposed to soil and house dust into which some EDCs such as PBDEs may leach [64] and because they have a tendency to consume items other than conventional foodstuffs. To date, such animals have been studied little but, as with domestic animals and humans, economic interests may cause this to change if effects are demonstrated.

## Other commercially important species

While not, typically, the subject of conventional veterinary treatments, the commercial importance of some invertebrate groups should not be forgotten. Honey bees are clearly of great economic importance and populations in Europe and North America appear to be under threat from a combination of factors, possibly including exposure to endocrine disrupting, agricultural chemicals [65]. Other invertebrate groups of commercial interest include marine and freshwater shellfish. Much of the interest in freshwater has concerned effects of EDCs in sewage on fish populations but lower profile, invertebrate species are also affected. For example, freshwater pearl mussels, already under threat from over-exploitation, are further compromised by exposure to the anti-depressant drug fluoxetine (Prozac) present in sewage effluent discharged into rivers; exposure causes premature release of their larvae, with associated reductions in their survival [66]. Effects of tributyl tin, an anti-fouling agent used in marine paints, has long been recognised as a potent disruptor of dogwhelk reproduction [67] but larval stages of bivalve molluscs, some of which are significant commercial species, are highly sensitive to this EDC [68]. While large, mammalian species generally have a higher profile and are the subject of greater management and veterinary inputs, the commercial and biological significance of more lowly species should not be underestimated and neither should the potential adverse effects on them of environmental pollutants.

## Understanding EDC actions

As indicated above, exposure of both species and individuals to pollutants is highly variable, depending on environment, diet and veterinary medical treatments, and multiple factors influence the animals’ responses. The occurrence of disruptions of reproduction and health that can be clearly attributed to the effects of pollutants is rare, reflecting the fact that effects are generally subtle, involving changes in gene expression and the structure and function of internal organs but not gross changes in health or reproductive performance. However, such subtle effects can be greatly exacerbated in animals subject to additional stressors; thus prediction of responses to exposure is difficult. For example, depending on the species and environment, stressors can be nutritional [69], osmotic [70] or thermal [71]. In each of these examples, the effect of the combination of insults (pollutant burden and other stressor) resulted in differences in tissue EDC burdens or enhanced susceptibility to the pollutants. Interactions between EDCs and other factors are poorly understood but may be a significant factor in animal health because subtle, underlying physiological disruptions that have no detectable effect in the healthy, unstressed, animal may become important when combined with other influences. Domestic animals can also suffer from such stressors and, in modern production systems, particularly social stressors [72] but the effects of such factors on responses to EDCs has been little investigated in domestic animals at this time.

## The future

Since EDCs are not causing widespread, acute, animal health issues, they may seem unimportant. However, it is important to remember that many of their effects are very subtle, and include changes in expression of particular genes, in fetal organ development and in animal reproductive performance and health, none of which are readily detected. Evidence of health effects in humans include increased incidences of testicular abnormalities and reduced fertility [22]. However, while the body of *circumstantial evidence* indicating a probable role of EDCs in these effects is large and rapidly growing, generally, it is virtually impossible to demonstrate, directly, *causal links* between EDC exposure and effect.

Setting aside the overarching threat that EDCs may pose to animal and human health and ecosystem sustainability, in practice, effects of EDCs may be of concern because of small effects on the long term health and productivity of domestic animals [39], particularly if they are acting in conjunction with other adverse influences. Thus, subtle changes in fetal development may result in a chronic and equally subtle, adverse effects in adult growth, reproduction or health which may be economically significant but are not readily corrected by management or veterinary intervention.

The extent to which animals are exposed to EDCs, and therefore the associated risk, is likely to depend on species and management practice e.g. with increasing costs of artificial fertiliser, the application to land of processed wastes such as sewage sludge and green waste compost is increasing. Production systems are also moving to the extremes; while low input systems are increasingly favoured in hill and upland areas, this in turn results in more intensive production and increased pesticide use in areas of high quality land and perhaps higher densities of animals with associated social stresses. Each trend has implications for the rate of EDC exposure and for the effects of exposure, as indicated above.

It is easy to focus entirely on the negative consequences associated with the production and use of EDCs but it is important to recognise the need for a more balanced view. Taking the use of analgesics as an example, while they may have disruptive effects in fetal development, this disbenefit must be considered in the light of the enormous potential benefits of their use. Similarly, while pesticides, herbicides, components of plastics and many other everyday products contain EDCs which may pose an insidious threat to animal health and productivity, they also provide massive benefits in terms of food production and human and animal health. Thus, optimising their production and use may not be easy and will certainly require a better understanding of the rates of exposure to, and actions of, these chemicals.
